# Adaptation of muscle activation after patellar loading demonstrates neural control of joint variables

**DOI:** 10.1038/s41598-019-56888-9

**Published:** 2019-12-30

**Authors:** Filipe O. Barroso, Cristiano Alessandro, Matthew C. Tresch

**Affiliations:** 1 Department of Physiology, Northwestern University, Chicago, IL 60611 USA; 2 Department of Biomedical Engineering, Northwestern University, 2145 Sheridan Road,, Evanston, IL 60208 USA; 3 Department of Physical Medicine and Rehabilitation, Northwestern University, Chicago, IL 60611 USA; 4 Shirley Ryan AbilityLab, Chicago, IL 60611 USA

**Keywords:** Neurophysiology, Spinal cord, Central pattern generators

## Abstract

We evaluated whether the central nervous system (CNS) chooses muscle activations not only to achieve behavioral goals but also to minimize stresses and strains within joints. We analyzed the coordination between quadriceps muscles during locomotion in rats before and after imposing a lateral force on the patella. Vastus lateralis (VL) and vastus medialis (VM) in the rat produce identical knee torques but opposing mediolateral patellar forces. If the CNS regulates internal joint stresses, we predicted that after imposing a lateral patellar load by attaching a spring between the patella and lateral femur, the CNS would reduce the ratio between VL and VM activation to minimize net mediolateral patellar forces. Our results confirmed this prediction, showing that VL activation was reduced after attaching the spring whereas VM and rectus femoris (RF) activations were not significantly changed. This adaptation was reversed after the spring was detached. These changes were not observed immediately after attaching the spring but only developed after 3–5 days, suggesting that they reflected gradual processes rather than immediate compensatory reflexes. Overall, these results support the hypothesis that the CNS chooses muscle activations to regulate internal joint variables.

## Introduction

In order to affect movement, vertebrate muscles must generally act through joints. Structures within a joint, such as articular cartilage or ligaments between bones, translate muscle forces into skeletal movements^1^. Excessive stresses or strains within these internal joint structures can lead to joint pain and injury. It would therefore make sense for the CNS to regulate the state of these structures, choosing muscle activations to avoid excessive stresses or strains.

The close relationship between joint mechanics and limb movement makes it difficult to evaluate neural control of internal joint states. Manipulations affecting joint stresses and strains will usually also affect limb movements and so neural adaptations observed after the manipulation could reflect control of either set of variables. We have recently identified an experimental model using the rat knee joint that permits the separate study of task performance and internal joint variables^2,3^. Rats have a deep patellar groove in the femur^4^ that restricts mediolateral movement of the patella throughout the range of motion. The opposing mediolateral patellar forces produced by vastus lateralis (VL) and vastus medialis (VM) therefore do not result in distinct knee torques but, if these forces are unbalanced, could cause damaging patellofemoral loading or even patellar dislocation. VL and VM in the rat therefore have antagonistic actions on the internal joint variable of mediolateral patellar forces but have redundant actions on tibial movement. This separation between task actions (joint torques, tibial movement) and internal joint actions (mediolateral patellar forces) is not as clear in humans because of the increased patellar mobility when the limb is in full extension^5^, making the rat an attractive model for experiments examining the neural control of internal joint variables.

Using this rat model, we recently showed that the adaptation strategy used by the CNS following selective paralysis of a quadriceps muscle reflects a compromise between restoration of task performance (i.e. local joint angles) and regulation of internal joint variables (i.e. mediolateral forces on the patella)^2^. Although that work provided support for the neural control of internal joint variables, the muscle paralysis by nerve cut in those experiments is a large perturbation affecting both motor and sensory aspects of motor control^6–10^. Further, the effect of nerve cuts cannot be reversed to examine de-adaptation following muscle paralysis, making it difficult to interpret long term changes in muscle activation.

In the experiments described here, we therefore used a complementary perturbation paradigm to evaluate this hypothesis. We examined how the CNS adapted to the imposition of a lateral load on the patella. If the CNS regulates the state of internal joint structures, we would predict that the ratio between VL and VM should be decreased after application of this patellar load so as to reduce the lateral component of patellar forces, and therefore compensate for the imposed external load. Experiments using patellar taping to apply patellar loads in humans have shown inconsistent effects on VM and VL electromyography signals (EMGs)^11,12^. The consequences of patellar loading in humans, however, are potentially complex because of patellar mobility^5^. Further, those studies all examined short term effects of patellar taping, because of the difficulties in performing long term EMG recordings in humans. However, changes in EMGs in response to patellar loading, might only develop over multiple days, similar to the progressive adaptation observed in response to changes in musculoskeletal properties^13–15^. We therefore exploited the relative simplicity of the patellar mechanics in the rat and the ability to perform long term recordings of EMGs to examine whether patellar loading alters the balance of activation between VM and VL during locomotion and whether this alteration is consistent with the regulation of internal joint stresses and strains.

## Results

### Adaptation of VL/VM activation ratio after patellar spring attachment and detachment

**Figure 1:**
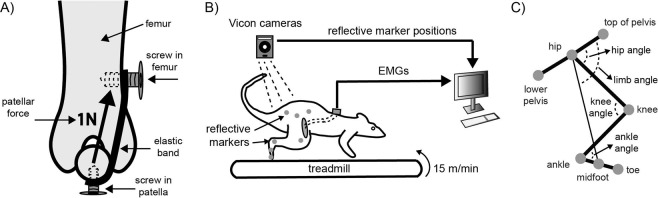
(**A**) Schematic illustrating how forces were applied to the patella. In an initial surgery, screws were implanted in the patella and lateral femur. After animals recovered to this implantation, a spring consisting of an elastic band was attached between the implanted screws. The distance between the screws was chosen so that the spring produced approximately 1N force, based on the stress-strain properties of the band. (**B**) EMGs and hindlimb kinematics were measured during treadmill locomotion. (**C**) Schematic illustrating landmarks at which retroreflective markers were placed on the hindlimb and the calculated joint angles.

**Figure 2:**
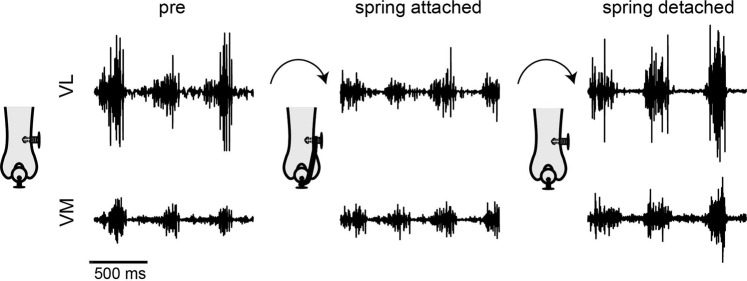
Example of VL and VM EMGs during treadmill locomotion before (pre), after attaching the spring, and after detaching the spring in one rat (rat 3 indicated in subsequent Figures).

We examined adaptation of quadriceps muscle activation to the imposition of a lateral force on the patella. This force was imposed by attaching a spring (an elastic band) between screws implanted in the patella and lateral femur (Fig. 1A). We then measured EMGs and hindlimb kinematics during treadmill locomotion (Fig. 1B,C). A representative example of VL and VM activations before attaching, after attaching, and after detaching the spring is illustrated in Fig. 2. The activation of VL decreased after the spring was attached whereas the activation of VM was relatively unaffected. This shift in the balance of activation between VL and VM is consistent with an increased net medial muscular force on the patella to compensate for the imposed lateral force on the patella caused by the spring. After removing the load by cutting the spring, activation of VL returned to levels similar to baseline, demonstrating that the reduction in VL activation reflected adaptation to the spring attachment. Activation of VM was relatively unchanged, although there was a modest increase in activation in this animal.

**Figure 3:**
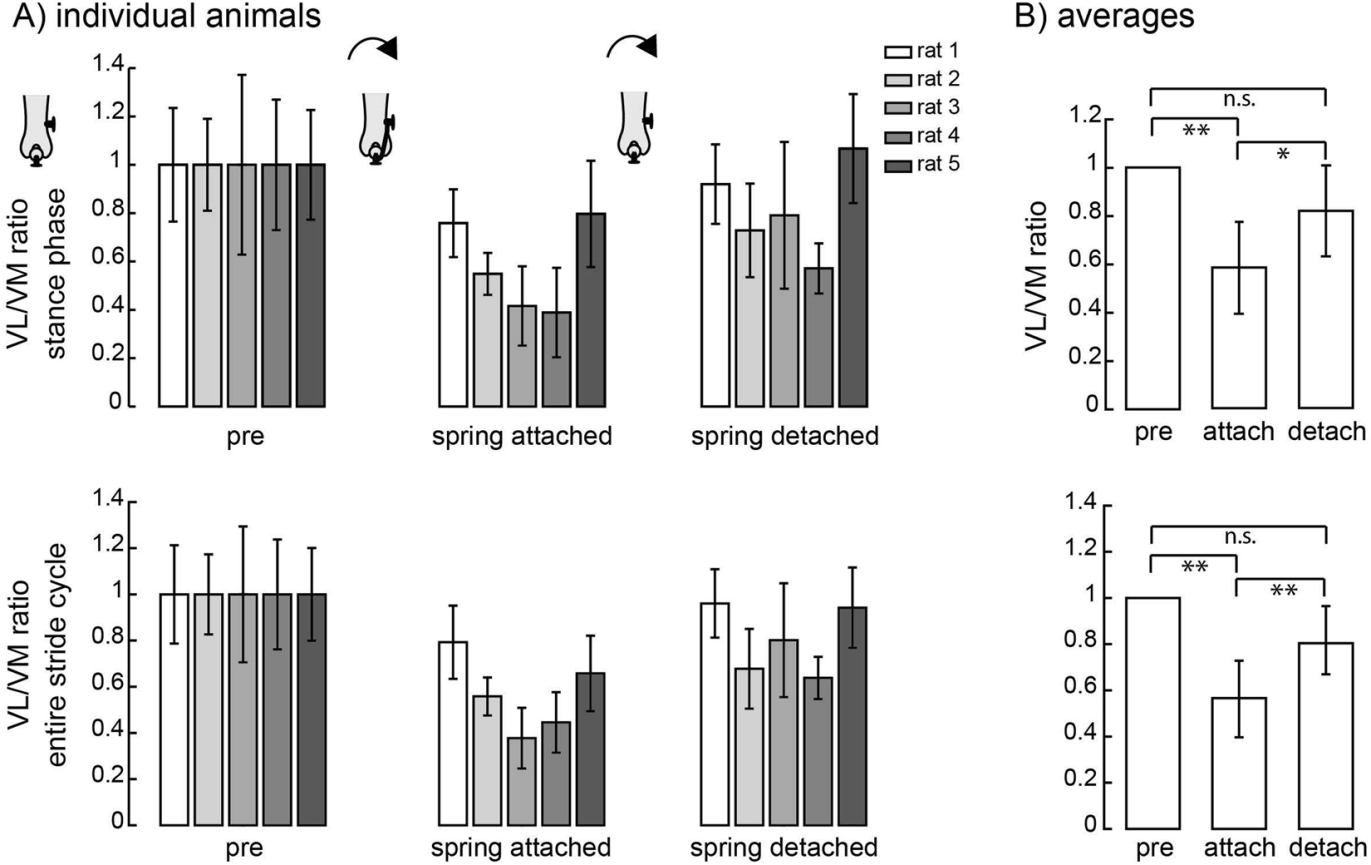
Upper panel. (**A**) VL/VM ratio during stance phase for each rat across spring attachment conditions. The VL/VM ratio during the baseline/pre-attachment condition was normalized to 1 for each animal. The ratio measured after attaching the spring and after detaching the spring are expressed as fractions of this baseline value. Note that there was no significant difference between the ratio between VL and VM measured early (3–5 days) and late (10–12 days) time points when the spring was attached/detached (p > 0.05); data was therefore combined across both days to calculate the mean values in the bar plots for each animal. Error bars indicate standard deviation across strides around each mean. Number of strides for each animal (N_s_) = 30/25/24/57/36 for pre-attached, N_s_ = 85/40/64/27/6 for attached, and N_s_ = 74/43/83/84/86 for detached conditions. (**B**) VL/VM ratios averaged across animals; error bars show standard deviations across animals around each mean. Lower panel. (**A**) VL/VM ratio across the entire stride cycle for each rat, across spring attachment conditions. (**B**) VL/VM ratio averaged across animals for the entire stride cycle. (p < 0.05*; p < 0.01**; p < 0.001***; n.s., no significant difference.).

The changes in the ratio between VL and VM measured during the stance phase of locomotion are shown in Fig. 3A (upper panel) for each individual animal. Across animals, there was a significant change in the balance of activation between VL and VM (Fig. 3B upper panel; ANOVA, p = 0.002). Post-hoc tests revealed that the ratio between VL and VM was significantly reduced when the spring was attached (p = 0.0099) as compared to baseline/pre-attachment values and was significantly increased when the spring was detached as compared to values after attachment (p = 0.016). There was no significant difference in the ratio measured before attaching and after detaching the spring (p = 0.19). Note that the pattern of changes in the VL/VM ratio across spring attachment conditions was consistent in each individual animal. Neither of the animals receiving sham surgeries in which no spring was attached showed this same pattern of changes (Supplementary Fig. 1), suggesting that the alterations illustrated in Fig. 3 reflected adaptation to the imposition of the patellar load rather than effects of surgical procedures. We also analyzed the ratio between VM and VL activation measured over the entire stride (Fig. 3 - lower panel) and found the same pattern of results (ANOVA, p = 0.0049): the ratio between VL and VM was reduced after spring attachment (p = 0.0069), and increased following detachment (p = 0.0096) to levels that were not different from pre-attachment levels (p = 0.071).

**Figure 4:**
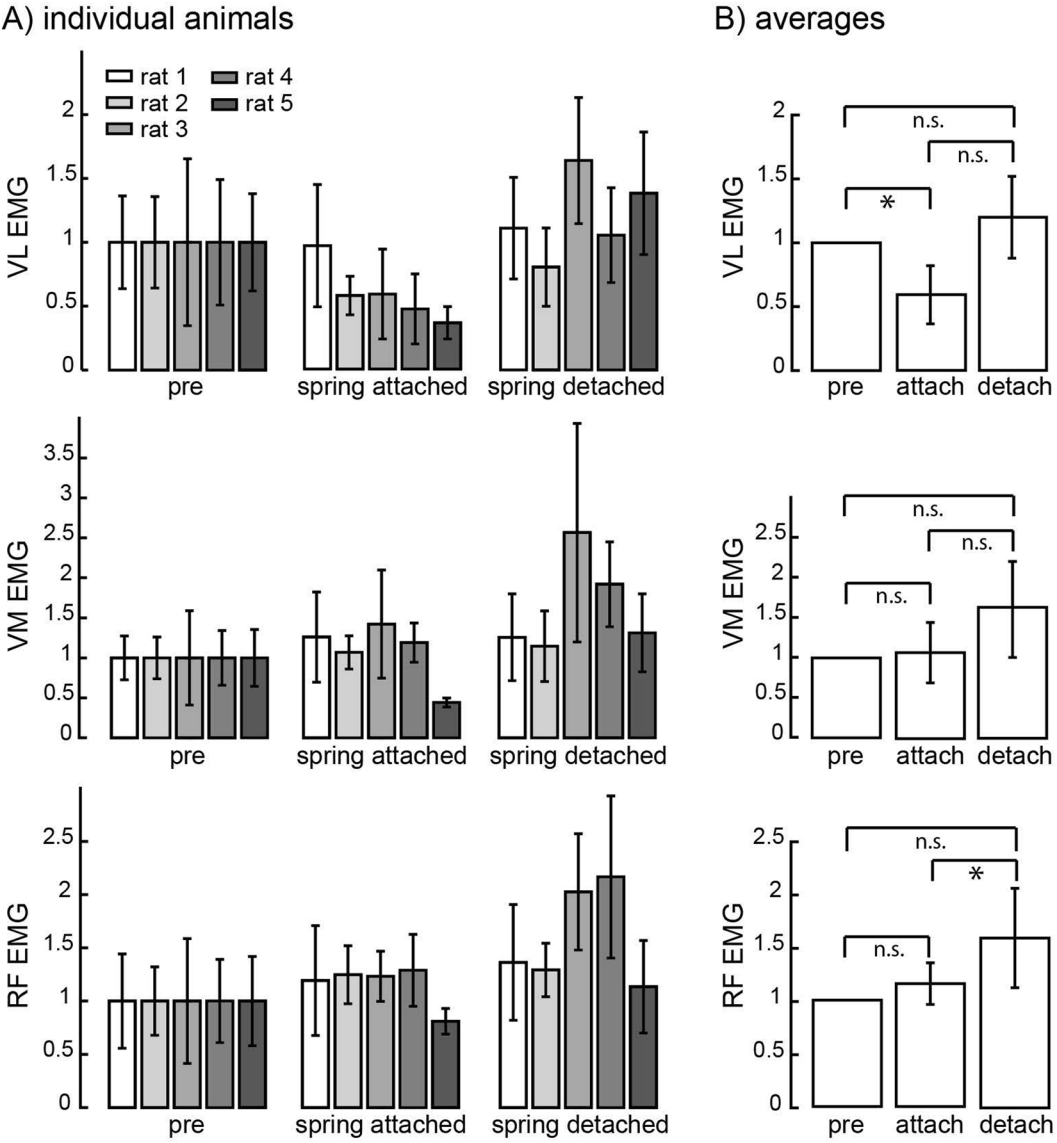
Activation of VL, VM, and RF across spring attachment conditions for individual animals (**A**) and averaged across animals (**B**). For VL and RF, N_s_ = 30/25/24/57/36 for pre-attached, N_s_ = 85/78/64/27/6 for attached, and N_s_ = 74/43/83/84/86 for detached conditions. The activation of each muscle during the baseline/pre-attachment condition was normalized to 1 for each animal. The number of observations was the same for VM except for the attached condition for rat2 for which the 40 strides (not 78) were observed because of a faulty connection on one day of recording. Conventions are the same as in Fig. 3.

The change in the ratio between VL and VM after attaching the patellar load was primarily due to changes in the activation of VL (ANOVA, p = 0.022; Fig. 4A,B top). VL activation was significantly reduced after attaching the spring (p = 0.029). There was no significant difference between VL activity after the spring was detached as compared to when the spring was attached (p = 0.053) or compared to baseline condition (p = 0.55). There was no significant difference in VM activation across spring attachment conditions (Fig. 4A,B middle; ANOVA; p = 0.064). Activation of another quadriceps muscle RF, however, was significantly different across conditions (Fig. 4A,B bottom; ANOVA, p = 0.036), showing a significant increase in activation levels after detaching the spring (p = 0.020); there was no significant change after attaching the spring (p = 0.64) or between baseline and detached conditions (p = 0.091). Sham control animals did not show similar patterns of VL, VM and RF activation across procedures (Supplementary Fig. 1).

### Minimal change in VL/VM ratio immediately after applying patellar load

**Figure 5:**
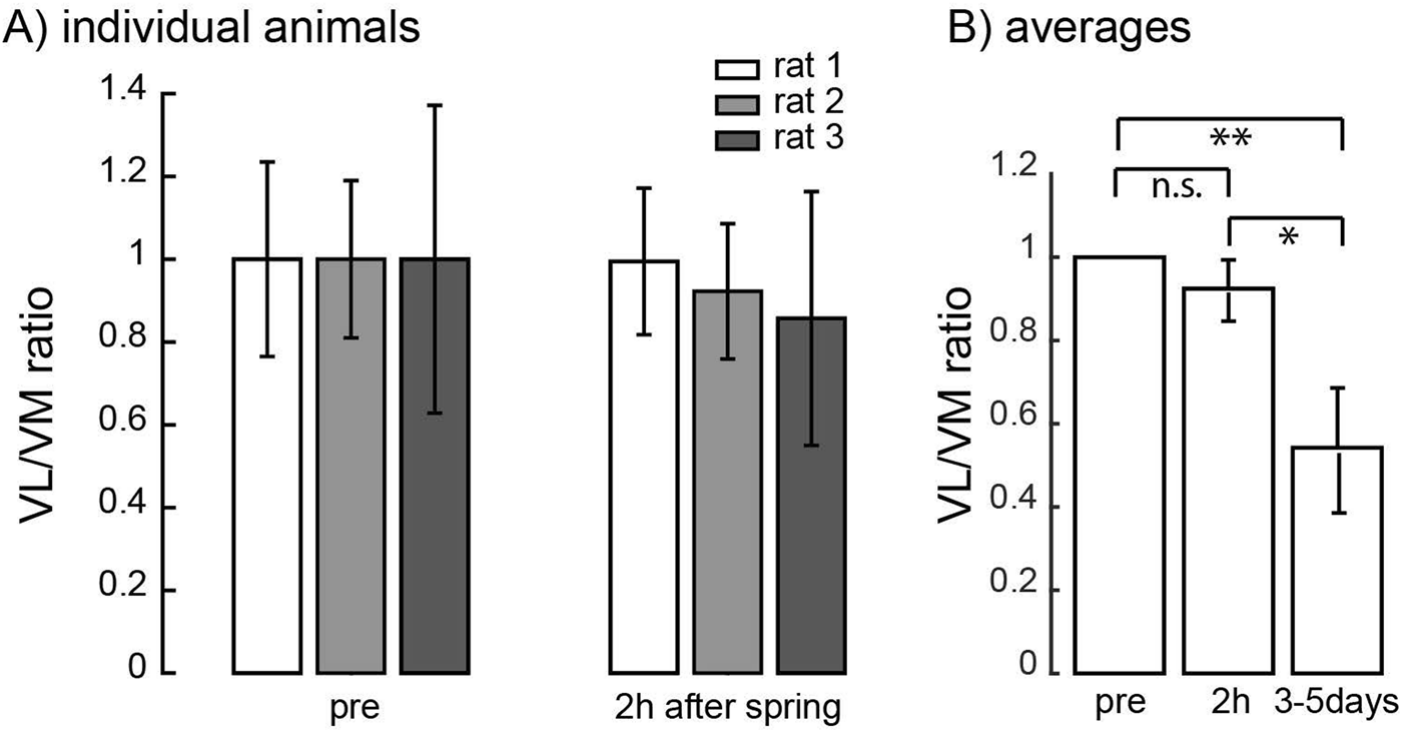
VL/VM ratio measured 2 hours after attaching the spring for individual animals (**A**) and averaged across animals (N = 3) (**B**). (**B**) also illustrates the VL/VM ratio for these same animals measured 3–5 days after attaching the spring. N_s_ = 30/25/24 for pre-attached, N_s_ = 24/32/23 for 2 h after spring attachment, and N_s_ = 42/40/40 for 3–5 days after spring attachment conditions. Conventions are the same as in Fig. 3.

Changes in the ratio between VL and VM after attaching/detaching the spring could reflect the action of reflexes monitoring patellar loads and altering VL/VM activations to minimize the net mediolateral forces on the patella. However, we found that changes in the VL/VM ratio only fully developed after several days of altered patellar loading (ANOVA, p = 0.0028; Fig. 5A,B). There was no significant difference in VL/VM ratio when measured 2 hours after attaching the spring (p = 0.47), whereas the ratio was significantly reduced a few days later compared to baseline (p = 0.006) and compared to 2 hours after attaching the spring (p = 0.0216). These results suggest that the alteration of VL and VM activation reflected a gradual adaptation process or gradual development of joint inflammation rather immediate reflex compensation.

### Effects of patellar spring on task performance and hindlimb kinematics

As illustrated in Fig. 1A, although the attached spring pulled laterally on the patella, it also pulled the patella proximally towards the hip. We verified in a terminal experiment that the attached spring produced a knee extension torque. The magnitude of the force at the distal tibia was 0.13 ± 0.05 N corresponding to a knee extension torque of 0.0045 ± 0.0016 Nm. Because of this knee extension torque, the spring attachment might be expected to alter joint kinematics during locomotion.

**Figure 6:**
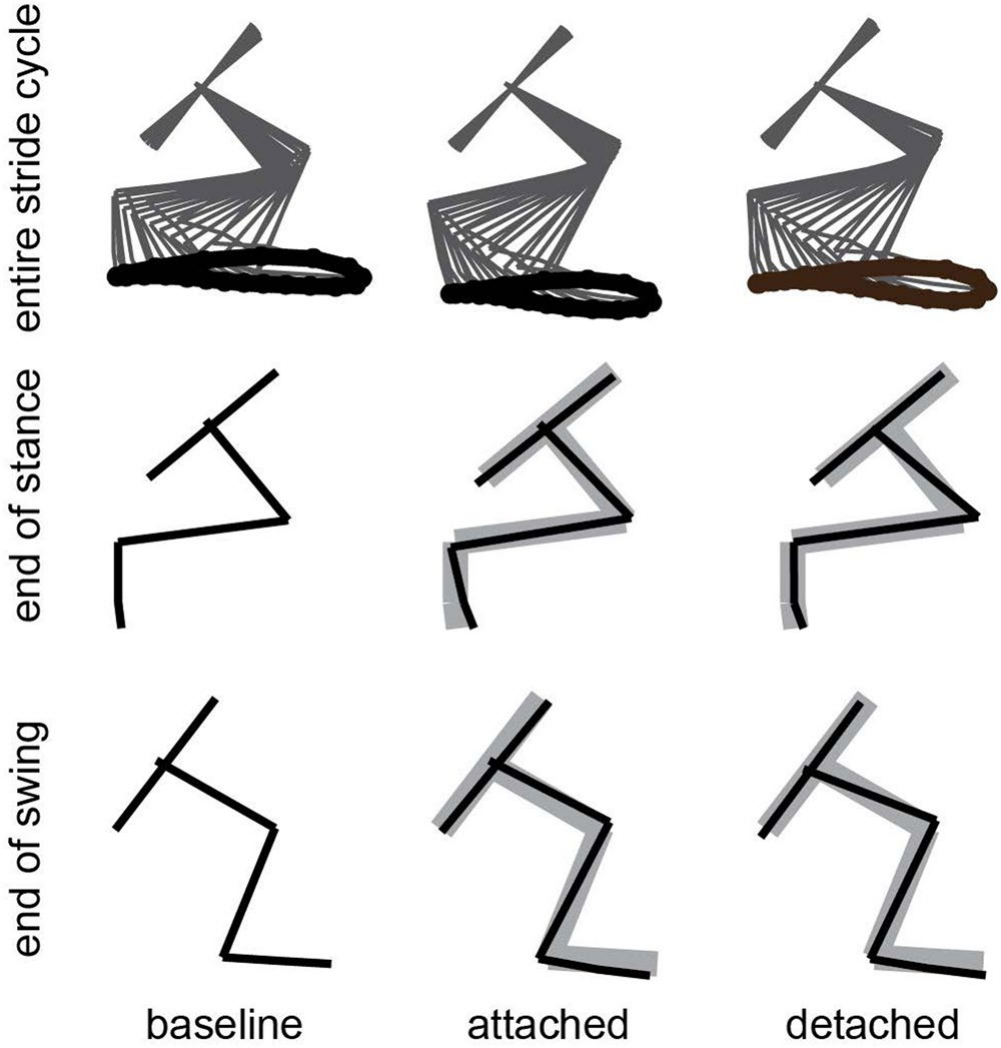
Hindlimb kinematics in baseline, spring attached, and spring detached conditions. Top traces show hindlimb kinematics across the stride cycle averaged across animals. The thick black trace indicates the trajectory of the toe marker. Middle traces show the hindlimb configuration measured at the end of the stance phase averaged across all animals. The thin black lines illustrate the hindlimb configuration for each experimental condition. The thick gray lines in the spring attached and spring detached conditions illustrate the limb configuration measured in baseline/pre-attachment condition, included for purposes of comparison. Bottom traces show the hindlimb configuration measured at the end of the swing phase, for each experimental condition.

**Figure 7:**
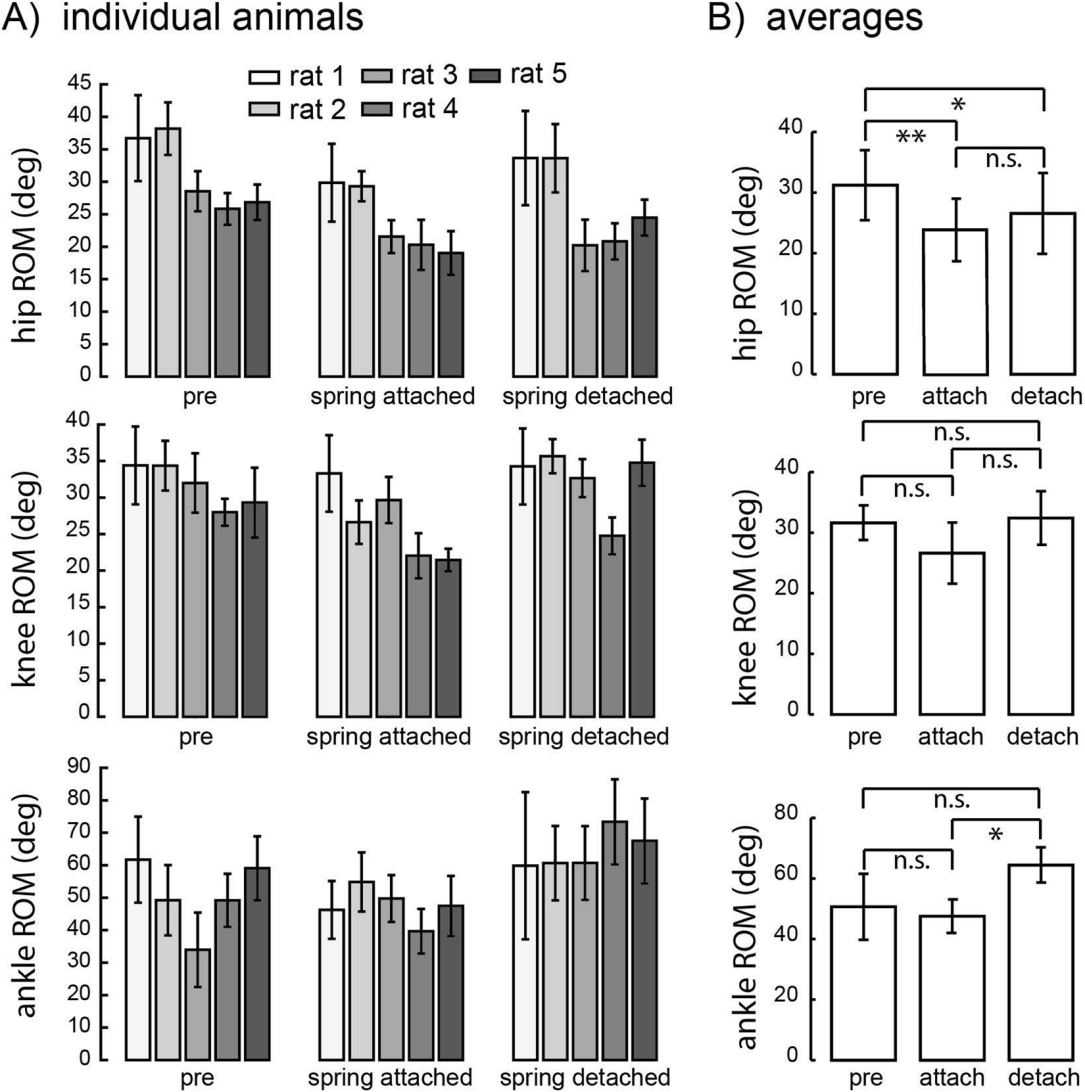
Changes in the range of motion for each local joint angle (hip, knee, ankle) within the sagittal plane for each animal (**A**) and averages across animals (**B**). N_s_ = 30/25/29/57/36 for pre-attached, N_s_ = 85/78/64/27/6 for attached, and N_s_ = 74/43/83/84/86 for detached conditions. Conventions are the same as in Fig. 3.

**Figure 8:**
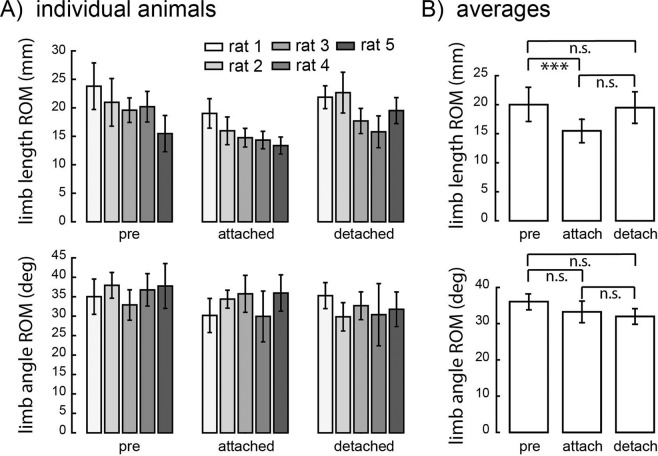
Changes in the range of motion for the global limb kinematics measures of limb angle and limb length. (**A**) shows values for individual animals; (**B**) shows values averaged across animals. N_s_ = 30/25/29/57/36 for pre-attached, N_s_ = 85/78/64/27/6 for attached, and N_s_ = 74/43/83/84/86 for detached conditions. Conventions are the same as in Fig. 3.

Figure 6 illustrates average limb kinematics across the stride cycle across all animals before, after attaching the patellar spring, and after detaching the spring (top traces in Fig. 6), as well as the limb configuration at the end of the stance phase (middle traces in Fig. 6) and at the end of the swing phase (bottom traces in Fig. 6). The range of motion for each individual joint was significantly altered across attachment conditions (Fig. 7; ANOVA; p = 0.0012 for hip; p = 0.049 for knee; p = 0.013 for ankle). At the hip, the range of motion was significantly reduced following spring attachment (p = 0.002) and remained reduced after detachment (pre vs. detached, p = 0.014; attached vs. detached, p = 0.63). At the ankle, there was a significant increase in range of motion only after detaching the spring (p = 0.017; pre vs. attached, p = 1; pre vs. detached, p = 0.090). At the knee, the pattern of changes was similar to that of the hip, but no specific post hoc comparison was significant (pre vs. attached, p = 0.061; attached vs. detached, p = 0.22; pre vs. detached, p = 1). Although both sham control animals showed a decreased range of motion at the hip after attaching the spring (Supplementary Fig. 2), they did not show a consistent decrease in range of motion at the knee nor did they show a consistent increase after spring detachment. We also examined measures of global limb kinematics (Fig. 8). There was no significant difference in the range of the limb vector angle across attachment conditions (ANOVA, p = 0.071). The range of limb vector length, however, was significantly altered (ANOVA, p < 0.001), being significantly decreased when the spring was attached (p < 0.001); there was no significant difference between baseline and detached (p = 1) or between attached and detached conditions (p = 0.056). Sham control animals showed increased limb vector length after attachment and detachment of the spring compared to baseline condition (Supplementary Fig. 2). On the other hand, sham control animals showed similar limb vector angle across attachment conditions (Supplementary Fig. 2).

Although there were significant changes in the ranges of motion of joints caused by the spring attachment, there were less consistent effects on the actual joint angles. We first analyzed local and global joint angles at the end of stance, when the consequences of altered quadriceps activation should be observable; the only significant change was a reduction in the knee angle (ANOVA, p = 0.0013; p = 0.0018) after detaching the spring. We also examined kinematics at the end of the swing phase; after spring attachment, there was a significant increase in limb vector angles (ANOVA, p < 0.001; p < 0.001). There were no significant differences across spring attachment conditions for all other joint angles (p > 0.05).

We also evaluated more standard measures of gait characteristics (Supplementary Fig. 3). The percent of the stride spent in stance was significantly affected (ANOVA, p = 0.0063), being reduced after attaching the spring (p = 0.0081); there was no significant change after detaching the spring (p = 0.51) or between pre and detached conditions (p = 0.12). There was no difference in the step height (ANOVA, p = 0.16) or stride duration (ANOVA, p = 0.49) across spring attachment conditions. Taken together, these results show that there were significant alterations in limb kinematics following spring attachment, consisting of a reduced range of motion in several joints and a reduction in stance phase duration. Sham controls showed very similar values of the percent of stride spent in stance across attachment condition conditions (Supplementary Fig. 2). On the other hand, sham controls did not show similar step height across procedures but walked with slightly increased duration of stride across conditions.

## Discussion

We found that animals adapted to imposition of a lateral force on the patella by altering the balance of activation between VL and VM. After attaching a lateral spring to the patella, animals reduced the ratio between VL and VM activation and this reduction was reversed after the spring was detached. These adaptations were not observed immediately after attaching the spring but developed over several days. There were also alterations in limb kinematics after attaching the spring, with reduced ranges of motion of joint angles and reduced percent of stance. Animals receiving sham surgeries did not show similar alterations in muscle activation or limb kinematics.

These results are consistent with the hypothesis that the CNS chooses muscle activations to regulate the internal stresses and strains within joints, consistent with our previous experiments examining adaptation to selective muscle paralysis^2^ and with other studies showing adaptation of VM and VL in response to patellar loading^16–19^. The reduction in the ratio between VL and VM activation suggests that the CNS altered the balance between these muscles in order to regulate the net mediolateral force on the patella. This reduction was observed consistently in each individual animal, further suggesting the important role of internal joint variables in the neural control of movement.

The change in the ratio between VL and VM after spring attachment was accomplished primarily by reduction of VL activation, with no significant change in the activation of either VM or RF. If the implanted spring pulled only laterally, this adaptation would not only reduce the lateral force on the patella but would also reduce the net knee torque, resulting in perturbed knee kinematics. However, as indicated in Fig. 1A and confirmed experimentally, the implanted spring pulled both laterally and proximally on the patella. The reduction of VL activation can therefore be interpreted as compensating for both actions of the spring, working to restore mediolateral patellar forces and net knee extension torques. Note that if the CNS only acted to maintain task performance, it could have reduced activation of VL, VM or RF to compensate for the increased knee torque. The fact that VL was selectively and consistently reduced provides strong support for the idea that the CNS controls joint stresses and strains.

The restoration of task performance was incomplete, however, as indicated by the persistent alterations in hindlimb kinematics observed after spring attachment. It is possible that this incomplete compensation was due to the relatively short adaptation periods examined here; our previous experiments showed that periods of at least one month were necessary for animals to achieve stable compensation strategies after VL paralysis^2^. We used the shorter two-week adaptation period in this study to minimize the chance of the spring detaching or breaking during the experiment. The incomplete restoration of task performance observed here might also reflect the fact that the knee extension torque produced by the spring was constant across the locomotor cycle. Compensating for this constant torque would require either precise alteration in the activation of multiple muscles throughout the stride (e.g. reducing knee extensors during stance, increasing knee flexors during swing) or altering the timing of muscles so they were active at atypical parts of the stride (e.g. tonic activation of knee flexors). Thus, although the imbalanced patellar forces could be compensated by simply changing the amplitude of a single muscle (reducing VL), compensating for the altered knee extension torques was more complex. It is also possible that the altered kinematics observed here reflect an appropriate compensation according to some unknown optimization criteria^20^ (e.g. minimizing changes in muscle commands and changes in limb kinematics). Finally, it is possible that the altered limb kinematics observed here reflected a nonspecific alteration in gait caused by discomfort induced by the spring attachment, as might be suggested by the reduced percent of the stride spent in stance. Future experiments examining longer adaptation periods, evaluating the role of inflammation in this adaptation, or using different patellar perturbations can help distinguish these possibilities.

In principle, mediolateral forces on the patella might be regulated by reflexes modifying the balance of activation between VL and VM. The observation here of minimal changes in muscle activation immediately after spring attachment might suggest that such ‘corrective’ reflex compensation^21^ was not responsible for the alterations in EMGs observed here. It is important to note, however, that the measurements taken a few hours after spring attachment could have been affected by analgesics administered following the procedures for attaching the spring that were absent at later time points. A gradual adaptation process in response to persistent alterations in mediolateral patellar forces is similar to the gradual adaptation process following injury or changes in musculoskeletal properties^13–15^. signaled directly to the CNS by sensory afferents within the knee joint^22^ or indirectly by proprioceptive afferents in quadriceps muscles^23^. Alternatively, the apparently gradual expression of altered muscle activations might reflect the gradual development of an inflammatory response to the altered patellar loads. The altered sensory feedback from this inflammation would then cause altered muscle activations, potentially through sensorimotor reflexes. Although reflexes from joint afferents have been shown to evoke motor responses^24,25^, the role of these afferents in specifying muscle activation remains unclear. Evaluating whether the absence of immediate compensation to altered patellar loads observed here reflects a gradual process of motor adaptation or a gradual development of inflammation will require further experiments.

Our results are related to clinical investigations examining patellofemoral pain (PFP) in humans^26,27^. Although many factors can contribute to PFP^27,28^, one common explanation is that PFP results from an imbalance between forces produced by VM and VL, usually explained by atrophy of VM or maladaptive coordination between these muscles^29–38^. One treatment for PFP is patellar taping to counter patellar maltracking or correct patellar loading^12,39,40^, similar to the perturbations performed in the current study. Studies have shown mixed effects of patellar taping on the timing and amplitude of quadriceps EMGs^11,12^, in contrast to the consistent decreased ratio between VL and VM found in our experiments. One reason for this difference might be the fact that we examined the effects of patellar loading over several days rather than after acute exposure as examined in humans. Our observation that alterations in VL/VM ratio developed gradually over persistent perturbation to patellar forces suggests that acute exposure might not be sufficient to evaluate the effects of altered patellar loading. Another potential explanation for the less consistent results in humans might be the greater complexity of patellofemoral mechanics^5,40^: the increased mobility of the patella in humans might require more complex coordination between quadriceps muscles than is necessary in quadrupeds such as rats.

There are several limitations of the current study. Further experiments examining a wider variety of patellar perturbations would help establish the robustness of these results; e.g. attaching springs in different directions or creating perturbations only during stance. Although the relatively short adaptation period used here ensured that the spring remained attached through the adaptation period, longer periods might have enabled animals to more completely compensate for the perturbation, for example, by restoring limb kinematics. Similarly, it would be useful to track the precise temporal evolution of the adaptation process over the first few days of spring attachment; although Fig. 5 shows that there was not an immediate alteration in EMGs after spring attachment, it would be useful to better characterize the time course of adaptation. Finally, the limited number of animals used here also might also limit the conclusions of this study, although the fact that the main result (reduction of VL/VM ratio) was consistently observed across all animals suggests that this is a robust finding.

Our work is also relevant for analyses considering how the nervous system handles redundancy in the musculoskeletal system^41–46^. Our work suggests that the apparent redundancy of the musculoskeletal system might be less significant when all variables determining muscle activation are considered^47^. In the context of the present study, VM and VL produce similar knee extension torques and so have redundant contributions to task performance. When the action of these muscles on mediolateral patellar forces is considered, however, this redundancy disappears; when both task performance and internal joint stresses are accounted for, the number of permitted activations of VM and VL is substantially reduced. Our results reinforce suggestions that understanding neural control strategies during behavior requires appreciating the full complexity of muscle actions, including muscle actions on internal joint stresses and strains.

## Methods

Seven Sprague Dawley rats (5 females, 2 males; weight 270–470 g; Charles River Laboratories International, Inc., Wilmington, MA) were tested in this study, in accordance with a protocol approved by Northwestern University Institutional Animal Care and Use Committee (Protocol Number IS00000628).

### Training on treadmill locomotion

Rats were acclimated to a motorized treadmill (Columbus Instruments, Columbus, OH) and trained for at least 7 days (30 minutes per day) to run for 5 consecutive minutes with a steady stable pattern of locomotion in the same general region of the treadmill, at speeds ranging from 10 m/min to 20 m/min.

### Implantation of EMG electrodes and bone screws

We implanted EMG electrodes in multiple hindlimb muscles, using techniques described previously^2,48^. Briefly, animals were anesthetized (2–3% isoflurane) and prepared for aseptic surgery. Pairs of electrodes were implanted into each quadriceps muscle (vastus lateralis, VL; vastus medialis, VM; rectus femoris, RF) and secured with knots so that an exposed region on each electrode was located within the muscle. Electrodes were then tunneled subcutaneously to a connector (Omnetics nano) placed in the back below the scapulae.

In the same surgical session, we also implanted two bone screws, one in the lateral femur and one in the patella (Fig. 1A). The patellar surface was exposed and a hole drilled in the center of the patella. We took care so that the drilled hole did not penetrate through to the opposite surface of the patella in order to avoid damage to patellofemoral surfaces (this was confirmed in post mortem examinations in each animal). The screw was then implanted so that ~0.5 mm length was exposed above the patellar surface and the screw was securely fixed within the bone. The other screw was implanted in the lateral surface of the femur and placed at a distance of ~17 mm from the patellar screw. Animals were given analgesics (meloxicam, buprenorphine) for 2 days after these surgical procedures.

### Recording baseline hindlimb EMGs and kinematics

Animals were allowed to recover after the implantation surgery and retrained on treadmill locomotion as necessary. We then recorded EMGs and hindlimb kinematics during locomotion in order to establish baseline performance prior to adding a load to the patella (Fig. 1B). We attached retroreflective markers to standardized landmarks on the rat hindlimb (Fig. 1C): iliac crest (top of the pelvis), greater trochanter (hip), caudal margin of ischium (lower pelvis), lateral condyle (knee), lateral malleolus (ankle), metatarsal-phalangeal joint (mid foot) and toe. The EMG connector was attached to a tethered cable connected to the acquisition system and the animal placed on the treadmill. The 3D position of each marker during locomotion was recorded using a four camera Vicon system at 200 Hz. EMG signals were amplified (1000×, A-M Systems Model 3500) and bandpass filtered (30–1000 Hz with 60 Hz notch). EMGs were digitized (2000 Hz, Vicon) and saved for subsequent offline analysis. Animals walked for at least one minute at the speed of 15 m/min.

### Evaluating effects of patellar spring attachment and detachment

After recording baseline behavior, we attached a spring between the screw placed in the patella and the screw in the lateral femur. A small incision (~2 cm) was made to expose the implanted screws and a spring (elastic band, diameter 6.4 mm) was attached between the two screws. The distance between the screws (~17 mm) was chosen so that the spring would apply approximately 1 N force, based on separate measurements of its stress-strain properties. The wound was then closed and animals were given analgesics for 2 days following these procedures. This spring attachment was successfully performed in 5 animals. In 2 additional animals the patellar screw came loose during the spring attachment procedure. We used these 2 animals as sham controls, in which we performed the same surgical procedures as the other 5 animals, but in which no spring was attached. Animals were given analgesics (meloxicam) for 2 days following these procedures.

We then repeated the measurements of EMGs and kinematics during locomotion. Animals walked for at least one minute at the speed of 15 m/min. We made these measurements in all animals at two time points: 3–5 days and 10–12 days after attaching the spring. In 3/5 animals we also measured EMGs and kinematics 2 hours after attaching the spring.

After the last measurement with the spring attached, we then detached the spring. This was a brief procedure, in which the implanted spring was exposed and cut. In all cases we observed that the implanted spring remained tightly stretched between the two screws before cutting it. For the 2 sham surgical control animals, we performed the same procedures without cutting any implanted spring. We repeated EMG and kinematic measurements for all animals at 3–5 days and 10–12 days after detaching the spring. Animals walked for at least one minute at the speed of 15 m/min.

### Acute experiment to measure end point forces caused by the patellar spring

As illustrated in Fig. 1A, the implanted spring did not produce a pure lateral force on the patella but also pulled the patella proximally. This proximally directed force should in principle produce a knee extension torque. To evaluate this possibility, we performed a terminal experiment in one animal to measure the end point forces produced by the implanted patellar spring. Methods were similar to those described previously^49,50^. Briefly, the rat was anesthetized with ketamine/xylazine (80/20 g/kg). Bone screws were attached to the pelvis and clamped to immobilize the pelvis. The distal tibia was attached to a 6 axis force transducer (ATI Mini40) using bone screws implanted in the tibia. We attached the spring between the screw in the patella and the screw in the lateral femur. The force at the tibia was measured for a range of knee angles (57–100 degrees). We then cut the implanted spring and repeated the force measurements at the same knee angles. We subtracted the force measurements before and after cutting the spring at each angle to estimate the force at the tibia produced by the implanted spring. Knee extension torque was calculated by multiplying the magnitude of the force difference by the distance between the transducer attachment and the center of rotation of the knee joint.

### Analysis of kinematics and spatiotemporal parameters

All data were analyzed offline using custom software in MATLAB (Mathworks, Natick, MA). Foot strike and foot off events were identified from kinematic traces. Foot strike events were used to define the beginning and end of each locomotion stride. To reduce errors due to the differential movement of the skin and the bones, we calculated the position of the knee by triangulation using the hip, knee, and ankle markers^51,52^. Data were then resampled at each 1% of the locomotion stride, so that each stride consisted of 100 data points. For each rat and trial, we identified periods in which each animal walked steadily in the same general region of the treadmill (a minimum of 10 consecutive strides). From the position of the seven markers within the sagittal plane, we computed joint angles (hip, knee and ankle), limb angle (angle between the vector from the hip marker and the marker at the top of the pelvis and the vector from the hip marker and the marker on the mid foot, Fig. 1C), limb length (distance between hip and mid foot markers), as well as step height (maximum height of mid foot marker during each stride), stride duration (time between consecutive foot strikes), and percent stance phase (percentage of the stride cycle when the paw was in contact with the ground).

### EMG analyses

EMG signals were high-pass filtered (40 Hz) to attenuate motion artifacts^53^. Filtered signals were then de-meaned^54^, rectified and low-pass filtered at 30 Hz, resulting in EMG envelopes. Baseline noise from EMG envelopes was removed by subtracting the mean of minimum values across all strides. We carefully inspected raw EMGs and envelopes from all muscles to identify artifacts that were occasionally produced by cable movement. The few strides presenting these artifacts were discarded from analyses. All the other non-corrupted strides belonging to the selected periods of locomotion were used for analysis of EMG data. EMG envelopes were then resampled to obtain 100 data points for each stride.

We focused on activation of quadriceps muscles during the extension phase of locomotion when VL, VM, and RF are typically active. This analysis excluded any flexion related burst in these muscles. For each of the quadriceps EMG envelopes, we identified the burst of activity that typically started before foot strike and ended before foot-off. This identification was performed separately for each animal and was usually straightforward for these muscles with clear stance related bursts (see Fig. 2). We then integrated the activity during this period for each individual locomotor cycle. To confirm that the results observed here did not depend on the determination of extension phase, we also evaluated the activation of muscles integrated across the entire stride cycle.

### Statistical analysis

All statistical analyses were performed with linear mixed effect models in Matlab. Post hoc comparisons between specific groups were made using Bonferroni corrected t-tests. Distributions were inspected for normality and square root transformed when necessary for the non-negative EMG distributions examined here. We first evaluated whether there was a significant difference in the ratio between VL and VM measured early (3–5 days) and late (10–12 days) after attaching/detaching the spring. We performed a mixed model analysis of the ratio between VL and VM with fixed effects of spring attachment state (after attaching vs. after detaching) and time of measurement (3–5 days vs. 10–12 days), with random slopes and intercepts for each animal. We found that there was no significant main effect of time of measurement (p = 0.83) and no interaction between spring attachment state and time of measurement (p = 0.09). Based on this result, in all subsequent analyses we combined measurements at both time points in order to simplify statistical models and increase statistical power. We then evaluated whether the ratio between VL and VM (normalized to the pre-attachment value) varied with spring attachment state, using a mixed model with fixed effect of spring attachment (pre/attached/detached) and random slopes and intercept for each animal. We used the same model to evaluate whether the activation of VL, VM, and RF (normalized to their pre-attachment values) varied with spring attachment state and to evaluate whether the ratio between VL and VM changed after 2 h of spring attachment. Finally, we evaluated kinematic (range of motion of hip, knee, ankle, limb angle and limb length) and spatiotemporal measures (percent of stance phase, step height and duration of stride), using a mixed model with fixed effects of spring attachment state (pre/attached/detached) and random slopes and intercepts for each animal. We also evaluated whether the joint angles measured at either foot strike or foot off differed across spring attachment states, using the same mixed model structure.

## Supplementary information

Supplementary information.
