# Author Correction: Adaptation of muscle activation after patellar loading demonstrates neural control of joint variables

**DOI:** 10.1038/s41598-021-95069-5

**Published:** 2021-08-16

**Authors:** Filipe O. Barroso, Cristiano Alessandro, Matthew C. Tresch

**Affiliations:** 1 Department of Physiology, Northwestern University, Chicago, IL 60611 USA; 2 Department of Biomedical Engineering, Northwestern University, 2145 Sheridan Road, Evanston, IL 60208 USA; 3 Department of Physical Medicine and Rehabilitation, Northwestern University, Chicago, IL 60611 USA; 4 Shirley Ryan AbilityLab, Chicago, IL 60611 USA

Correction to: *Scientific Reports* https://doi.org/10.1038/s41598-019-56888-9, published online 30 December 2019

The original version of this Article contained an error in Reference 2, which was incorrectly given as:

Alessandro, C., Rellinger, B. A., Barroso, F. O. & Tresch, M. C. Adaptation after vastus lateralis denervation in rats suggests neural regulation of joint stresses and strains. *Elife* 1–18, https://doi.org/10.1101/312488 (2018).

The correct reference is listed below:

Alessandro, C., Rellinger, B. A., Barroso, F. O. & Tresch, M. C. Adaptation after vastus lateralis denervation in rats suggests neural regulation of joint stresses and strains. *eLife* **7**, 38,215, https://doi.org/10.7554/eLife.38215 (2018).

The original Article has been corrected.

