# Supplementary material for: Adaptation of muscle activation after patellar loading demonstrates neural control of joint variables
Source: Sci Rep. 2019 Dec 30;9:20370. doi: 10.1038/s41598-019-56888-9 (PMC6937258; doi:10.1038/s41598-019-56888-9)
Supplement: Supplementary file 1 — Supplementary information. [file 41598_2019_56888_MOESM1_ESM.pdf]

**Supplementary Information for: Adaptation of muscle activation after patellar loading demonstrates neural control of joint variables**

Filipe O. Barroso[1], Cristiano Alessandro[1], Matthew C. Tresch[1,2,3,4*]

sham controls

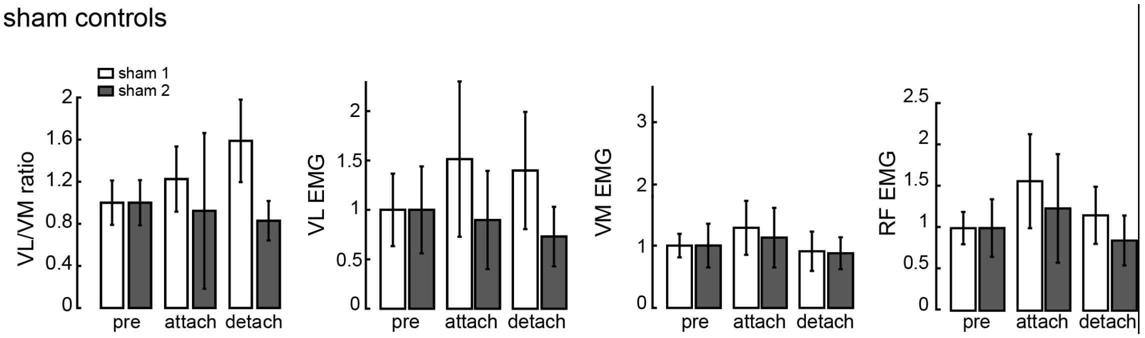


Supplementary Figure 1. Changes in VL/VM ratio, VL, VM, and RF activation for sham control animals in which the same surgical procedures were performed but no spring was attached to (or detached from) the patella.

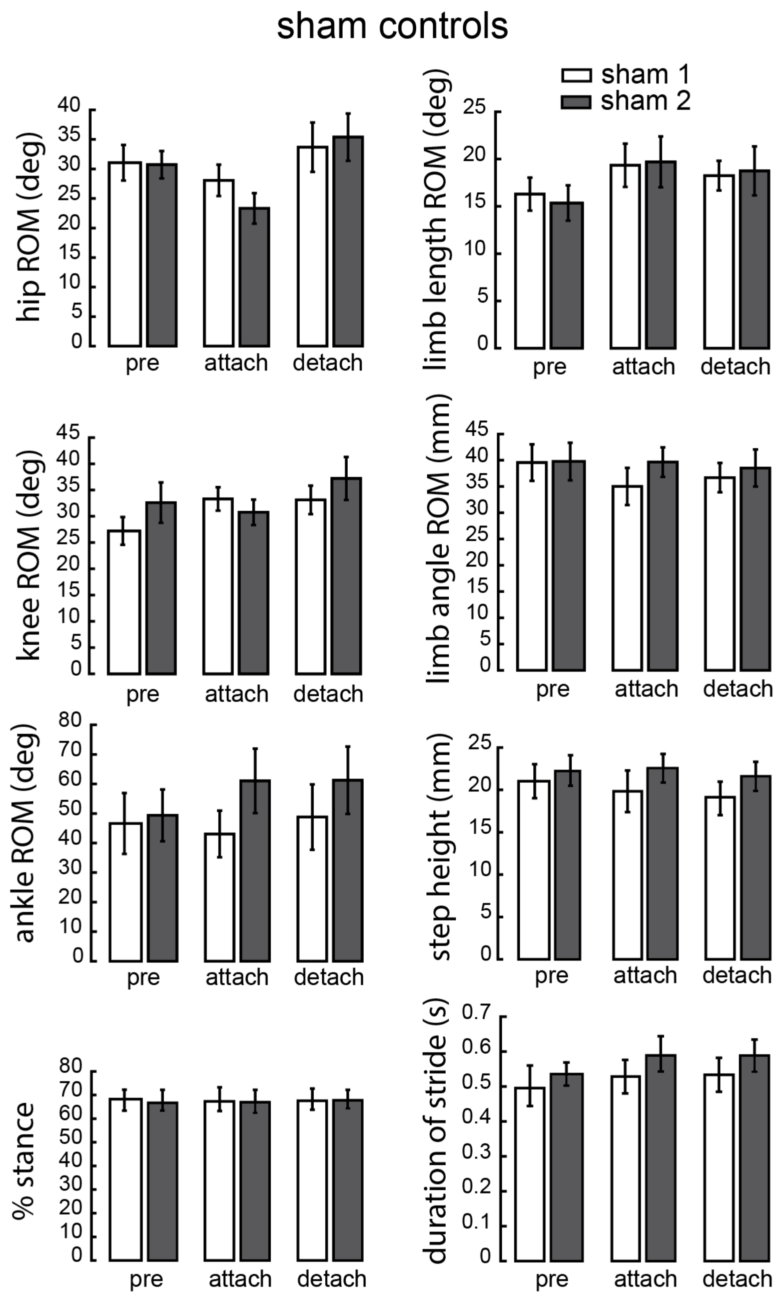


Supplementary Figure 2. Changes in limb kinematics and spatiotemporal parameters for sham control animals.

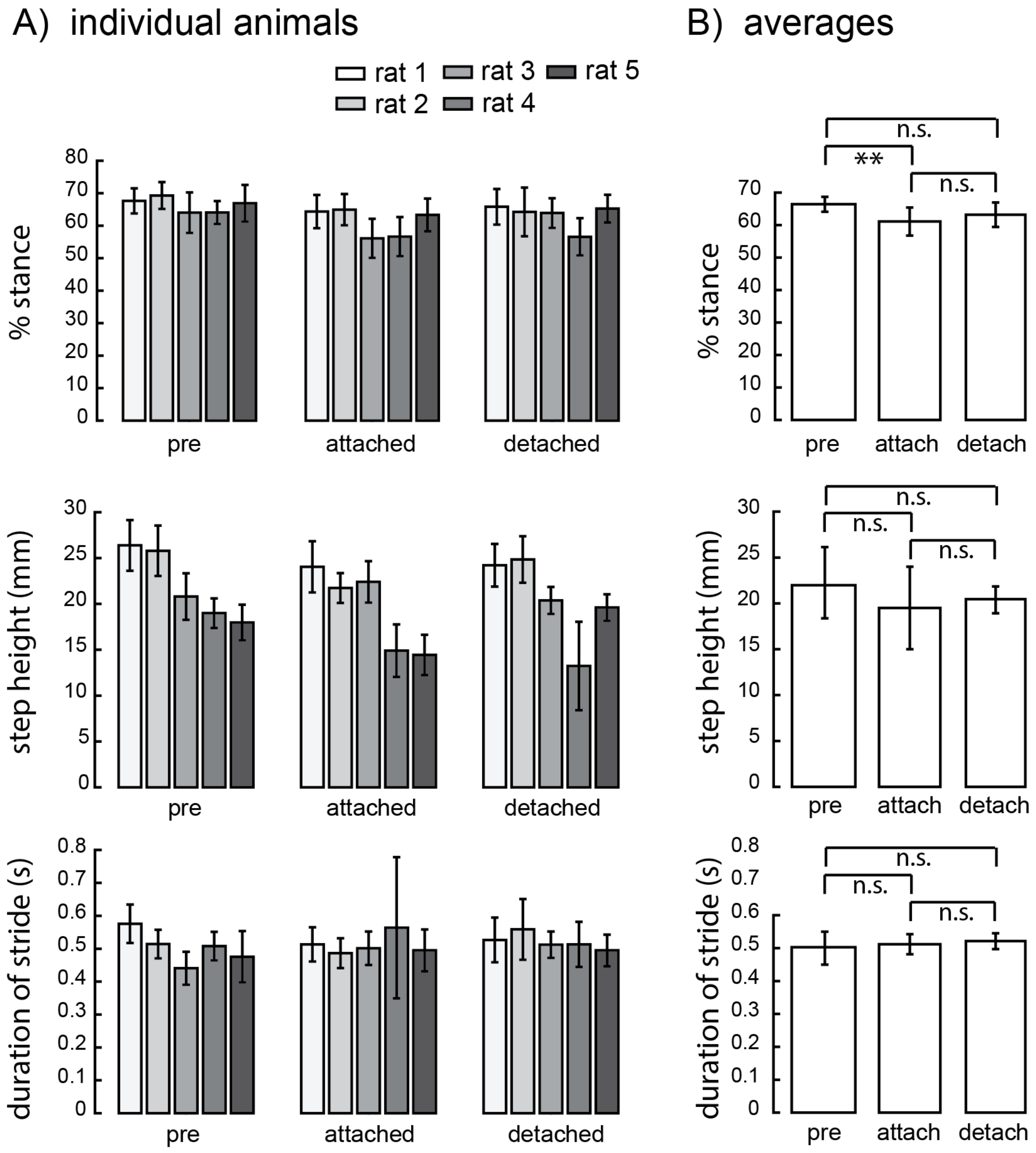


Supplementary Figure 3. Changes in spatiotemporal parameters. (A) shows values for individual animals; (B) shows values averaged across animals. $N_s$ = 30/25/29/57/36 for pre-attached, $N_s$ = 85/78/64/27/6 for attached, and $N_s$ = 74/43/83/84/86 for detached conditions. Conventions are the same as in Figure 3.
